# Plectin protects podocytes from adriamycin‐induced apoptosis and F‐actin cytoskeletal disruption through the integrin α6β4/FAK/p38 MAPK pathway

**DOI:** 10.1111/jcmm.13816

**Published:** 2018-09-06

**Authors:** Yongliang Ni, Xin Wang, Xiaoxuan Yin, Yan Li, Xigao Liu, Haixin Wang, Xiangjv Liu, Jun Zhang, Haiqing Gao, Benkang Shi, Shaohua Zhao

**Affiliations:** ^1^ Department of Geriatrics Qilu Hospital of Shandong University Jinan Shandong China; ^2^ Department of Urology Shandong Provincial Third Hospital Jinan Shandong China; ^3^ Department of Urology Tengzhou Central People's Hospital affiliated to Jining Medical College Xintan Road 181 Tengzhou China; ^4^ Department of Traditional Chinese Medicine Yankuang Group General Hospital Zoucheng China; ^5^ Department of Urology Qilu Hospital of Shandong University Jinan China; ^6^ Department of Urology Yankuang Group General Hospital Zoucheng China; ^7^ Key Laboratory of Cardiovascular Proteomics of Shandong Province Qilu Hospital of Shandong University

**Keywords:** apoptosis, F‐actin cytoskeleton, FAK, integrin α6β4, p38 MAPK pathway, plectin, podocyte

## Abstract

Podocyte injury is an early pathological change characteristic of various glomerular diseases, and apoptosis and F‐actin cytoskeletal disruption are typical features of podocyte injury. In this study, we found that adriamycin (ADR) treatment resulted in typical podocyte injury and repressed plectin expression. Restoring plectin expression protected against ADR‐induced podocyte injury whereas siRNA‐mediated plectin silencing produced similar effects as ADR‐induced podocyte injury, suggesting that plectin plays a key role in preventing podocyte injury. Further analysis showed that plectin repression induced significant integrin α6β4, focal adhesion kinase (FAK) and p38 MAPK phosphorylation. Mutating Y1494, a key tyrosine residue in the integrin β4 subunit, blocked FAK and p38 phosphorylation, thereby alleviating podocyte injury. Inhibitor studies demonstrated that FAK Y397 phosphorylation promoted p38 activation, resulting in podocyte apoptosis and F‐actin cytoskeletal disruption. In vivo studies showed that administration of ADR to rats resulted in significantly increased 24‐hour urine protein levels along with decreased plectin expression and activated integrin α6β4, FAK, and p38. Taken together, these findings indicated that plectin protects podocytes from ADR‐induced apoptosis and F‐actin cytoskeletal disruption by inhibiting integrin α6β4/FAK/p38 pathway activation and that plectin may be a therapeutic target for podocyte injury‐related glomerular diseases.

## 1 INTRODUCTION

Podocytes are highly differentiated glomerular epithelial cells and an integrated part of the glomerular barrier that plays a key role in preventing proteinuria. Podocyte injury is an early step in the pathogenesis of various glomerular diseases; thus, podocytes are excellent targets for therapeutic agents. Among the various pathophysiological mechanisms underlying podocyte injury, apoptosis and F‐actin cytoskeletal disruption are two critical pathological phenomena. Increasing evidence indicates that podocyte apoptosis plays an important role in the pathogenesis of proteinuria and the progression of chronic nephropathy.^1^, ^2^ Additionally, the F‐actin cytoskeleton has been shown to be essential for maintaining the special morphology of podocyte foot processes. F‐actin cytoskeletal disruption in podocytes results in foot process effacement and is associated with the pathogenesis of proteinuria, which is common among a spectrum of chronic kidney diseases.^3^, ^4^, ^5^ Therefore, elucidating the mechanisms underlying podocyte apoptosis and F‐actin cytoskeletal disruption is critical for the development of promising therapies for glomerular diseases.

Adriamycin (ADR)‐induced nephrosis is a commonly used animal model of podocyte injury as ADR can induce apoptosis and F‐actin cytoskeletal disaggregation in podocytes. We previously identified dozens of proteins that are differentially expressed between the isolated glomeruli of rats with ADR‐induced nephropathy and those of normal control (NC) rats (data not shown here) in a proteome analysis via isobaric tags for relative and absolute quantification (iTRAQ). Among these differentially expressed proteins, a cytoskeletal linker protein known as plectin caught our attention because of its versatility and significant suppression in the glomeruli of ADR‐treated rats. In addition to functioning as a linker, plectin plays a crucial role in cellular processes involving actin cytoskeleton dynamics^6^, ^7^; moreover, plectin can function as a scaffolding platform for signalling molecules, such as integrin α6β4^8^, ^9^ and focal adhesion kinase (FAK),^10^, ^11^ which is an intriguing new facet of its functional repertoire that has recently attracted attention from researchers. FAK is a key mediator of integrin signalling among different cellular functions in a variety of cells. Following activation by integrins, FAK undergoes autophosphorylation, forms a complex with other cellular proteins and triggers downstream signalling through its kinase activity or scaffolding function.^12^, ^13^ FAK and downstream p38 MAPK signalling have been shown to play vital roles in ADR‐induced podocyte apoptosis and F‐actin cytoskeletal remodelling.^4^, ^14^

Given that plectin is severely depleted in ADR‐treated glomeruli, this study aimed to evaluate its effects in ADR‐induced podocyte apoptosis and F‐actin cytoskeletal rearrangement and explore its relationships with integrin α6β4 and FAK.

## 2 MATERIALS AND METHODS

### 2.1 Antibodies and reagents

Antibodies specific for WT1, synaptopodin, desmin, phospho‐Y1494 integrin, integrin α6β4 and plectin were obtained from Abcam (Cambridge, UK), and antibodies that recognize glyceraldehyde‐phosphate dehydrogenase (GAPDH), phospho‐p38 MAPK, total p38 MAPK, phospho‐Y397 FAK and cleaved caspase‐3 were obtained from Cell Signaling Technology (Beverly, MA). Antibodies specific for FAK and Bax were obtained from Santa Cruz Biotechnology (Santa Cruz, CA). ADR was purchased from Sigma‐Aldrich (St. Louis, MO). pEGFP‐N1‐plectin plasmids, plasmids containing mutant β4 cDNA and empty vectors (mock) were obtained from Biosune Biotechnology (Beijing, China). Small interfering RNA (siRNA) specific for plectin (siPlectin) and scrambled siRNA were custom designed and synthesized by Sigma (St. Louis, MO). FAK inhibitor 14 was purchased from Tocris Bioscience (Bristol, UK), and the p38 inhibitor SB203580 was purchased from MedChem Express (Monmouth Junction, NJ).

### 2.2 Animals

Twenty male Sprague Dawley rats (200 g ± 20 g) were purchased from Shandong PengYue Laboratory Animals (Shandong, China). Rats were housed in a temperature‐controlled room with a 12‐hour light/12‐hour dark cycle and given free access to food and water. These rats were randomly divided into two groups (n = 10 per group): the ADR group and NC group. ADR‐induced nephropathy was achieved by a single injection of 7.5 mg/kg ADR (0.75 mg/mL in normal saline) via the tail vein. No rat died during the experiment. At the end of the 4th week after ADR injection, all rats were killed. Before death, body weight was recorded, and urine was collected from each rat in metabolic cages to determine 24‐hour urine protein (UP) and 24‐hour urine volume (UV). Blood was collected from the abdominal aorta, and serum was prepared via centrifugation and stored at −20°C. The 24‐hour UP level was determined by the pyrogallol red‐molybdate method (Cobas 6000C501; Roche: Basel, Switzerland). Levels of blood urea nitrogen (BUN) and serum creatinine (SCr) were measured by an Automatic Analyzer (7600‐020; Hitachi, Tokyo, Japan). The upper pole of the kidney was removed and fixed in 4% formaldehyde for routine histological examination and immunohistochemical study. Renal cortical tissues were separated from the remaining kidney and stored in liquid nitrogen for subsequent protein measurements. All animal experiments complied with the ARRIVE guidelines, were carried out in accordance with the National Institutes of Health guide for the Care and Use of Laboratory Animals (NIH Publications No. 8023, revised 1978) and were approved by the Ethics Committee on the Care and Use of Laboratory Animals in Qilu Hospital of Shandong University in Jinan, China.

### 2.3 Cell culture and treatment

The mouse podocytes used in this study were conditionally immortalized cells established by Dr. Peter Mundel (Harvard Medical School, Boston, MA). The podocytes were cultured in RPMI 1640 medium containing 10% foetal bovine serum (Gibco, Grand Island, NY), 100 U/mL penicillin, 100 mg/mL streptomycin and 10 U/mL mouse recombinant interferon‐γ (R&D Systems, Minneapolis, MN) at 33°C. To induce differentiation, we thermoshifted the cells to 37°C and incubated them in interferon‐free medium for 14 days. The podocytes differentiated into large cells with several small branches. All the cell culture dishes used herein were coated with type I collagen (Sigma‐Aldrich), and the cultured cells never reached 90% confluency.

To assess ADR‐induced podocyte injury and investigate plectin expression, we treated the podocytes with ADR at concentrations of 0.5 or 1.0 μg/mL for 6, 12 and 24 hours. For plectin overexpression, we performed transient transfection with Lipofectamine 2000 (Invitrogen: Waltham, MA) according to the manufacturer's instructions. Podocytes at 70%‐80% confluence were transiently transfected with pEGFP‐N1‐plectin plasmids or empty constructs for 6 hours and then cultured normally for 42 hours. To silence plectin expression, we transiently transfected the podocytes with siPlectin or scrambled siRNA at a final concentration of 20 nmol/L for 72 hours using Lipofectamine 2000 (Invitrogen: Waltham, MA). We monitored gene silencing efficiency by Western blot and selected the cells with the most efficient gene silencing for subsequent experiments. Y1494 in the β4 subunit of integrin α6β4 was mutated into a phenylalanine residue using a QuikChange Site‐directed Mutagenesis Kit (Stratagene: La Jolla, CA). Vectors containing the mutant β4 cDNA were transfected into podocytes using Lipofectamine 2000 according to the manufacturer's instructions. The stable transfectants were subsequently pooled, and the subclones expressing mutant β4 subunits on their cell surfaces were isolated by fluorescence‐activated cell sorting. To inhibit FAK or p38, we pretreated the cells with FAK inhibitor 14 or SB203580 for 1 hour before stimulation as listed in Table 1.

**Table 1:** Treatment for podocytes

Group name	Treatment for podocytes
NC group	Normal control podocytes
ADR group	Podocytes treated with adriamycin
ADR + plectin group	Restoring plectin expression in ADR‐treated podocytes via transfection with plasmids containing plectin cDNA
ADR + Mock group	The control of ADR + plectin group transfecting podocytes with empty vector
NC + siPlectin group	Repressing plectin expression in podocytes via transfection with siPlectin
NC + Scramble group	The control of NC + siPlectin group transfecting podocytes with scramble RNA
NC + siPlectin + β4 mutant group	The vectors containing the mutant β4 cDNA were transfected into podocytes before transfection with siPlectin
NC + siPlectin + Mock group	The control of NC + siPlectin + β4 mutant group
NC + siPlectin + FAK inhibitor group	Pretreating the podocytes with FAK inhibitor 14 before transfection with siPlectin
NC + siPlectin + p38 inhibitor group	Pretreating the podocytes with SB203580 before transfection with siPlectin
NC + siPlectin + DMSO group	The control of NC + siPlectin + FAK inhibitor group and NC + siPlectin + p38 inhibitor group

### 2.4 Immunoblotting

The podocytes were washed with cold PBS and lysed with RIPA buffer containing a proteinase inhibitor cocktail (Roche) and phosphatase inhibitors. Total protein was extracted from frozen renal cortical tissue samples using RIPA AQ3 lysis buffer (Beyotime Institute of Biochemistry, Shanghai, China). The cell lysates were separated on 12%, 10% or 8% gels and probed with the indicated primary antibodies. The proteins were subsequently detected by enhanced chemiluminescence (Pierce: Waltham, MA). The blocking buffer used for phospho‐immunoblotting contained 5% (w/v) BSA.

### 2.5 Real‐time PCR analysis

Total RNA was extracted from the podocytes using TRIzol (Omega Bio‐Tek: Norcross, GA), and cDNA was synthesized using a First‐strand cDNA Synthesis Kit (Fermentas, Burlington, Ontario, Canada). Real‐time quantitative PCR (qPCR) was performed in a 20 μL reaction mixture prepared with RealMaster Mix and SYBR Green (Tiangen, Beijing, China). The qPCR programme comprised the following steps: 95°C for 1 minutes followed by 35 cycles of 95°C for 5 seconds, 58°C for 15 seconds, and 68°C for 20 seconds. Each sample was analysed in triplicate, and GAPDH was used as an endogenous control. The following plectin primers were used in the study: forward: 5′‐GCACAAGCCCATGCTCATAGA‐3′ and reverse: 5′‐CAGGAGCCGTGTAACTCCC‐3′; amplicon size: 112 bp. The following GAPDH primers were used in the study: forward: 5′‐GACAACTTTGGCATCGTGGA‐3′ and reverse: 5′‐ATGCAGGGATGATGTTCTGG‐3′; amplicon size 135 bp.

### 2.6 Flow cytometry for apoptosis analysis

After treatment, the podocytes were stained with fluorescein‐isothiocyanate‐ labelled Annexin V and propidium iodide according to the manufacturer′s instructions (BioLegend: San Diego, CA) and analysed by flow cytometry on an FACSAria III flow cytometer (Becton Dickinson, Franklin Lakes, NJ).

### 2.7 ROS measurement

Dichlorodihydrofluorescein diacetate (DCFH‐DA) was used to detect the ROS level of podocytes. In time‐course experiments, exponentially growing podocytes were treated with ADR (0.5 μg/mL) for 0, 1, 2, 4, 6 and 12 hours. In mitochondrial protective agent experiments, podocytes were treated with ADR (0.5 μg/mL) and ADR (0.5 μg/mL) + MitoTEMPOL (10 μmol/L) for 12 hours. After incubation, the cells were washed with PBS (PH 7.4). Following stained with DCFH‐DA (Beyotime Biotechnology, Shanghai, China) for 20 minutes at 37°C, the cells were detected and analysed by flow cytometry (CytoFLEX; Beckman: Urbana, IL). The level of ROS generation was analysed by FlowJo software (FlowJo, LLC: Ashland, OR).

### 2.8 Immunofluorescence staining

The cells were fixed in 4% paraformaldehyde for 30 minutes at room temperature and then incubated with 1% BSA (with 0.3% Triton X‐100) for 60 minutes. The nuclei were stained with 4′,6‐diamidino‐2‐phenylindole (DAPI). For F‐actin staining, the cells were stained with FITC‐labelled phalloidin for 60 minutes at room temperature before staining with DAPI. Fluorescence was detected by laser scanning confocal microscopy (Leica, Wetzlar, Germany).

### 2.9 Renal histological investigation

Renal tissue specimens were fixed in a 4% formaldehyde solution for 48 hours, dehydrated in graded ethanol solutions, and then embedded in paraffin before being sectioned at a thickness of 5 μm. Morphological analysis of glomerular injury was conducted by light microscopy following haematoxylin and eosin staining.

### 2.10 Immunohistochemical analysis

Five‐micrometer‐thick serial sections of kidney tissue were incubated with the appropriate primary antibody overnight at 48°C before incubation with the appropriate secondary antibodies for 25 minutes at room temperature. The sections were then treated with peroxidase‐marked streptavidin/peroxidase before examination under an Olympus microscope (model BX‐51; Tokyo, Japan). We used a semiquantitative scoring system^15^, ^16^ to grade the intensity of the abovementioned immunoreactions. In each glomerulus, positively stained cells were scored according to their staining intensities, which were determined using the following scale: 0 (no staining), +1 (weak but detectable staining), +2 (moderate staining) and +3 (intense staining). Five areas in each slide were evaluated under a microscope at a magnification of 40×. An H‐score was calculated for each tissue sample by multiplying the percentages of cells in each intensity category by the corresponding staining intensities and then adding these products together. The calculation was performed using the following formula: H‐score = ∑(Pc × *s*), where *s* represents the intensity score, and Pc is the corresponding cell percentage. All immunohistochemical staining was independently assessed by two blinded pathologists.

### 2.11 Electron microscopy

Kidney cortical tissue samples were separated into 1‐μm‐thick blocks on ice and then immediately placed in 3% glutaraldehyde for 2 hours at 48°C before being post‐fixed with 1% osmium tetroxide for 1 hour, stained with 2% aqueous uranyl acetate, and dehydrated in graded ethanol solutions. After infiltration and polymerization, ultrathin sections were prepared, stained with uranyl acetate and lead citrate, and examined under an H‐800 transmission electron microscope (Hitachi Electronic Instruments, Tokyo, Japan).

### 2.12 Statistical analysis

Statistical analyses were performed with SPSS 13.0 (SPSS Inc., Chicago, IL). Data were analysed by Student's *t* test or one‐way ANOVA followed by Student‐Newman‐Keuls post hoc tests. All statistical tests were two‐sided and a *P* value < 0.05 was considered statistically significant for all tests.

## 3 RESULTS

### 3.1 ADR down‐regulated plectin expression and induced podocyte injury

**Figure 1:**
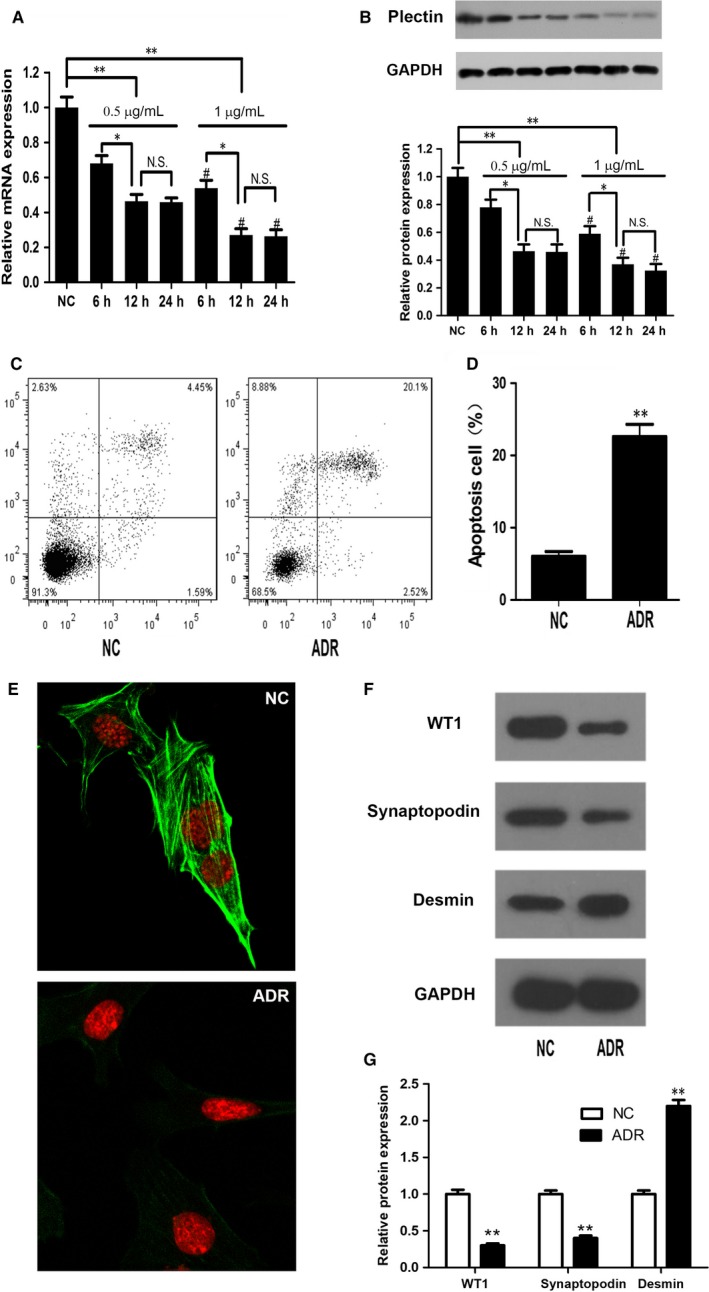
ADR suppressed plectin expression and induced podocyte injury. A‐B, Changes in plectin mRNA and protein expression in podocyte treated with ADR for the indicated times and at the indicated doses. *P < 0.05 and **P < 0.01. N.S., not significant. #*P* < 0.05 1 μg/mL vs 0.5 μg/mL ADR‐treated podocyte at the same time‐point. C‐D, Flow cytometry analysis showed that apoptosis was significantly increased in ADR‐treated podocyte compared with NC podocyte. **P < 0.01 vs the NC group. E, Immunofluorescence staining with FITC‐labeled phalloidin showed that the F‐actin cytoskeleton was disorganized in the ADR group compared with the NC group. F‐G, Western blot analysis showed that WT1 and synaptopodin expression levels were significantly decreased and that desmin expression levels were increased in ADR‐treated podocyte compared with NC podocyte. **P < 0.01 vs the NC group. Data shown are representative of five independent experiments (n = 5). The ADR‐treated podocyte in C‐G were treated with 0.5 μg/mL ADR for 12 h. ADR, adriamycin; NC, normal control; FITC, fluorescein isothiocyanate; F‐actin, filamentous actin

We treated podocytes with 0.5 or 1 μg/mL ADR for 6, 12, or 24 hours and then assessed plectin expression by qPCR and Western blot. We found that ADR down‐regulated plectin mRNA and protein expression in a concentration‐ and time‐dependent manner in treated cells compared with control cells (Figure 1A,B). However, there was no difference in plectin expression between the cells treated with ADR for 12 hours and those treated for 24 hours. We chose to treat podocytes with 0.5 μg/mL ADR for 12 hours in subsequent experiments (ADR‐treated group) because treatment with ADR at the indicated concentration and for the indicated time induced typical podocyte injury as well as significant decreases in plectin expression.

In vitro studies have shown that the actin cytoskeletal changes, such as F‐actin stress fibre loss, in cultured podocytes mimic the podocyte changes in vivo.^17^ Furthermore, rescue of the cytoskeletal disaggregation by genetic or chemical means in vitro correlates with protection from proteinuria in vivo.^18^ Therefore, we detected F‐actin cytoskeleton organization by fluorescein‐phalloidin staining to monitor podocyte injury. Immunofluorescence staining demonstrated that the F‐actin cytoskeleton, which appears as a dynamic network of intracellular proteinaceous structural elements in NC podocytes, was disorganized, collapsed and peripherally located in ADR‐treated cells (Figure 1E).

The second marker of podocyte injury examined in this study was the podocyte apoptosis rate, which is a critical determinant of the progression of proteinuria and renal failure. Flow cytometry analysis showed that apoptosis was significantly increased in the ADR group compared with the NC group (Figure 1C,D).

In this study, we also assessed the expression of several classical biomarkers that reflect podocyte injury. WT1 and synaptopodin are two specific podocyte markers whose expression is reduced when glomerular damage is present. Desmin has also been shown to be a sensitive marker of podocyte damage. WT1, synaptopodin and desmin protein levels were measured in podocytes by Western blot. The results showed that WT1 and synaptopodin expression levels were significantly reduced and desmin expression levels were increased in cells treated with 0.5 μg/mL ADR for 12 hours compared with control cells (Figure 1F,G).

### 3.2 ADR down‐regulated plectin expression through mitochondrial oxidative stress

**Figure 2:**
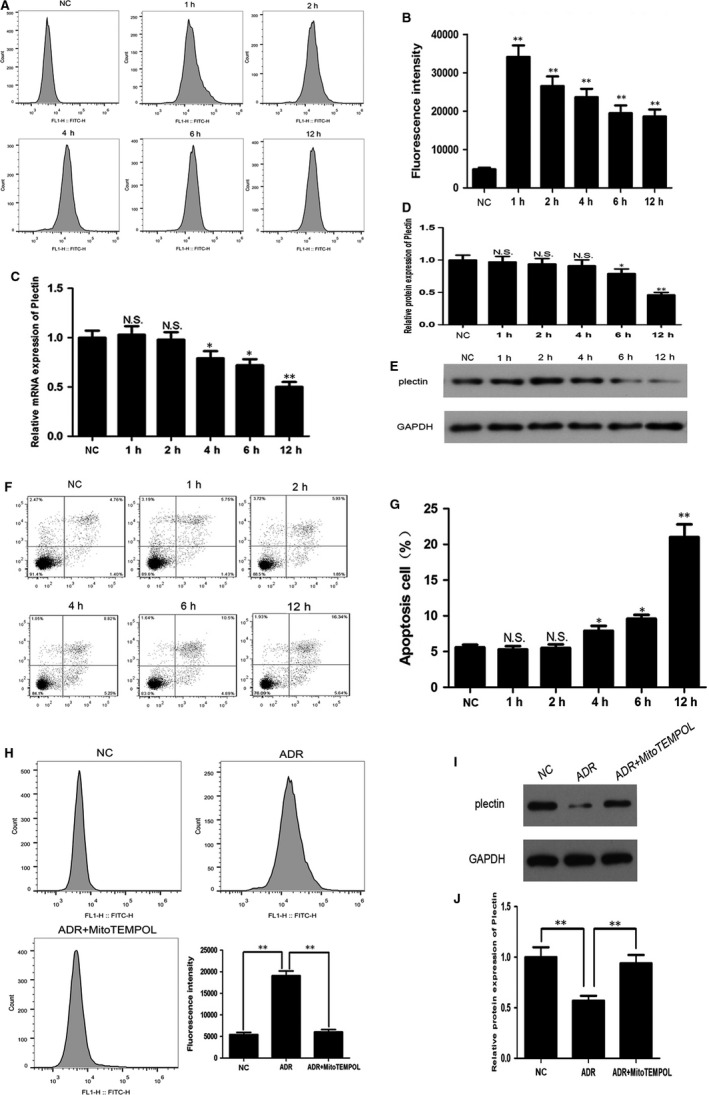
ADR suppressed plectin expression through mitochondrial oxidative stress. A‐B, Flow cytometry analysis showed that 0.5 μg/mL ADR treatment on podocyte for the indicated times caused an increase in ROS production. **P < 0.01 vs NC group. C‐E, Changes in plectin mRNA and protein expression in podocyte treated with 0.5 μg/mL ADR for the indicated times. *P < 0.05, **P < 0.01 and N.S., not significant vs NC group. F‐G, Flow cytometry analysis showed that 0.5 μg/mL ADR treatment on podocyte for 4 h caused an increase in apoptosis rate. *P < 0.05, **P < 0.01 and N.S., not significant vs NC group. H, Flow cytometry analysis showed that mitochondrial protective agent MitoTEMPOL (10 μmol/L) significantly decreased ROS production in ADR‐treated podocyte. I‐J, Western blot analysis showed that plectin expression was significantly increased in ADR + MitoTEMPOL group compared with ADR group. Data shown are representative of five independent experiments (n = 5). NC, normal control podocyte; ADR, podocyte treated with 0.5 μg/mL adriamycin for 12 h; ADR + MitoTEMPOL, podocyte treated with 0.5 μg/mL adriamycin and 10 μmol/L MitoTEMPOL for 12 h

Previous studies have shown that ADR caused mitochondrial dysfunction in podocytes, and the increased production of reactive oxygen species (ROS) by mitochondrial oxidative stress is one of the major manifestations.^19^, ^20^ In this study, we found that 0.5 μg/mL ADR treatment on podocytes for 1 hour caused significant increase in ROS production (Figure 2A,B), at which time‐point plectin mRNA and protein expression have not been down‐regulated (Figure 2C‐E), suggesting that mitochondrial damage preceded plectin down‐regulation. Mitochondrial protective agent MitoTEMPOL was added to the ADR‐treated podocytes and significantly decreased ROS production (Figure 2H). Plectin expression was significantly higher in ADR + MitoTEMPOL group compared with ADR group (Figure 2I,J), suggesting that mitochondrial oxidative stress is an important reason leading to plectin down‐regulation.

### 3.3 Restoring plectin expression prevented ADR‐induced podocyte injury

**Figure 3:**
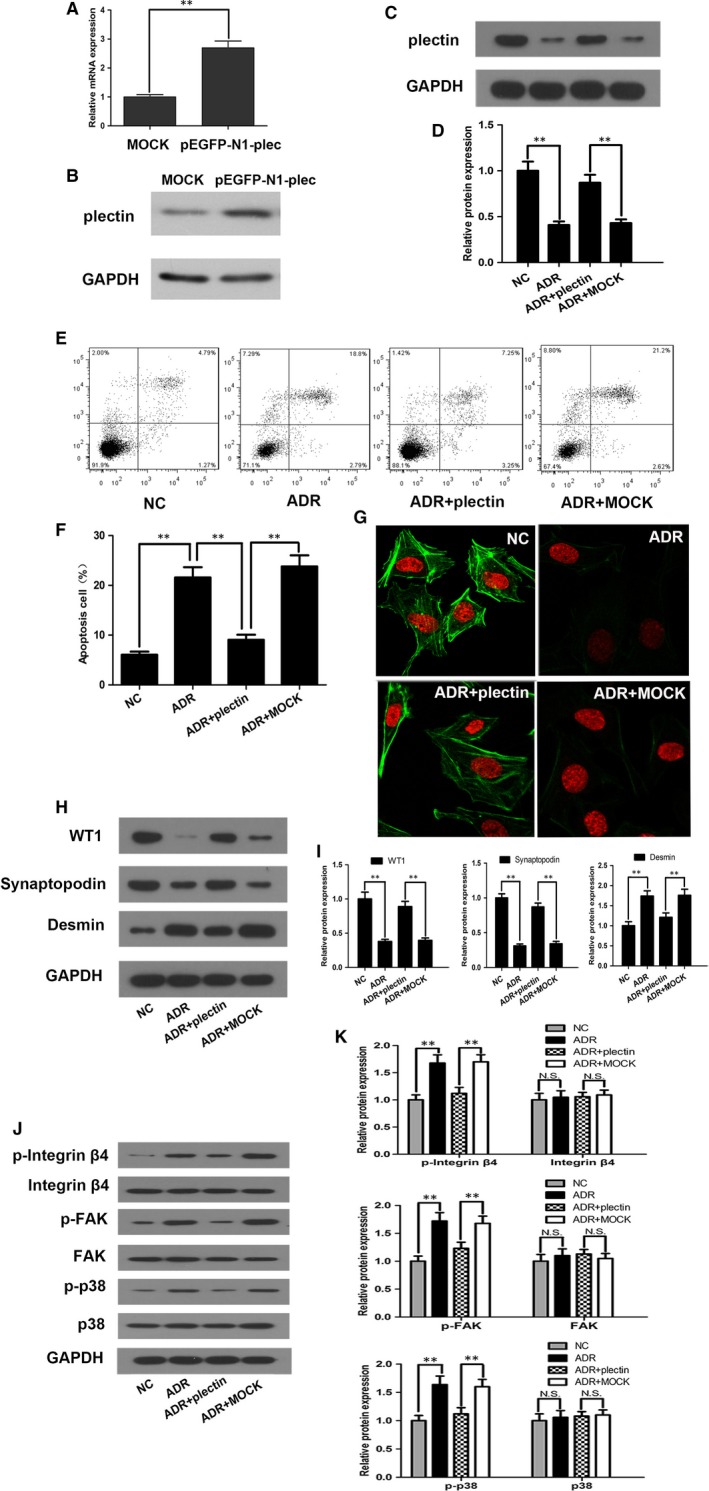
Restoring plectin expression prevented ADR‐induced podocyte injury. For the ADR group, podocyte was treated with 0.5 μg/mL ADR for 12 h. For the ADR + plectin group or ADR + MOCK group, podocyte was incubated with pEGFP‐N1‐plectin plasmids or empty vectors for 6 h and then cultured normally for 42 h, 0.5 μg/mL ADR was added 12 h prior to cell harvest. A‐B, Real‐time PCR and Western blot analysis showed that plectin expression was 2‐3 times of that in control group after transfecting pEGFP‐N1‐plectin plasmids into podocyte. C‐D, Western blot showed that plectin protein expression levels were significantly increased in the ADR + plectin group compared with the ADR and ADR + Mock groups. E‐F, Flow cytometry analysis showed that podocyte apoptosis was alleviated in the ADR + plectin group compared with the ADR and ADR + Mock groups. G, Immunofluorescence staining showed that restoring plectin expression in the ADR + plectin group ameliorated ADR‐induced F‐actin filament disruption. H‐I, Western blot showed that WT1 and synaptopodin protein expression levels were increased and that desmin protein expression levels were decreased in the ADR + plectin group compared with the ADR and ADR + Mock groups. J‐K, Western blot showed that integrin α6β4, FAK and p38 phosphorylation levels were higher in the ADR group than in the NC group. Restoring plectin expression in the ADR + plectin group suppressed integrin α6β4, FAK and p38 phosphorylation but had no effect on total integrin α6β4, FAK and p38 expression levels. Data shown are representative of three independent experiments (n = 3). **P < 0.01; N.S., not significant. ADR, adriamycin; F‐actin, filamentous actin; FAK, focal adhesion kinase; FITC, fluorescein isothiocyanate; NC, normal control

To determine the role of plectin in ADR‐induced podocyte injury, we restored plectin expression in ADR‐treated podocytes via transfection with plasmids containing plectin cDNA and then measured the subsequent changes. We found that plectin expression levels were significantly increased in the ADR + plectin group compared with the ADR and ADR + Mock groups, as demonstrated by Western blot analysis in Figure 3C,D. Flow cytometry assays, the results of which are presented in Figure 3E,F, showed that podocyte apoptosis was decreased in the ADR + plectin group compared with the ADR and ADR + Mock groups. Restoring plectin expression also mitigated the above mentioned ADR‐induced F‐actin cytoskeletal disarrangements, a finding that was also supported by our immunofluorescence staining results (Figure 3G). Western blot analysis showed that the expression levels of podocyte‐specific markers WT1 and synaptopodin were significantly enhanced but the expression level of desmin, a marker of injury, was substantially decreased by plectin restoration (Figure 3H,I). The above results demonstrated that restoring plectin expression significantly attenuated ADR‐induced podocyte injury, suggesting that plectin plays an important role in this pathological process.

### 3.4 Plectin suppression produced similar effects as ADR‐induced podocyte injury

**Figure 4:**
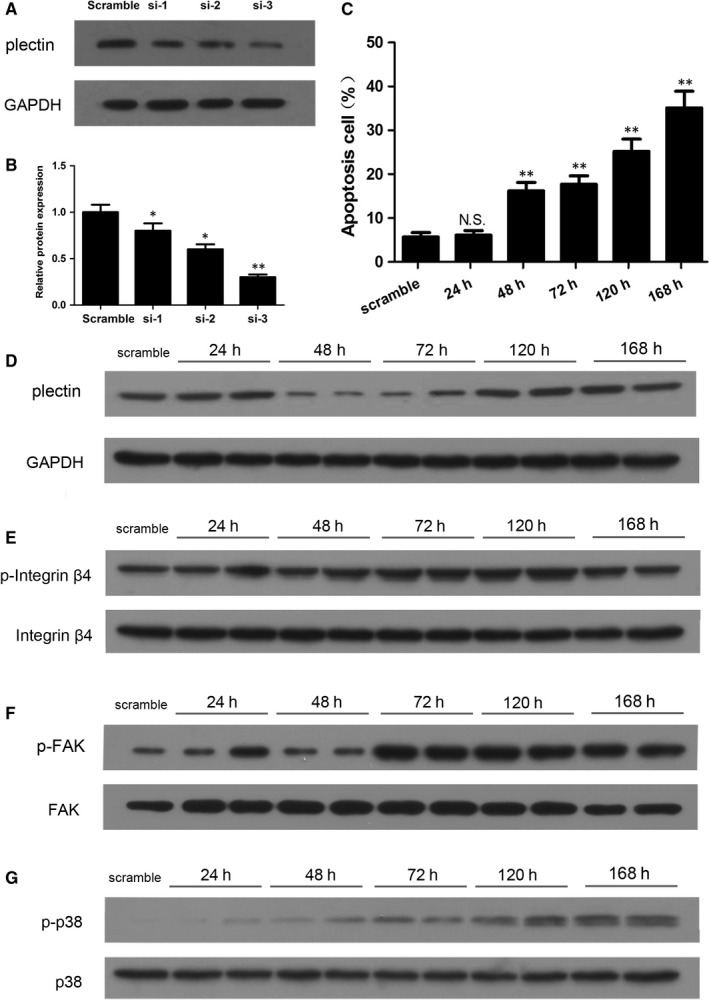
The time‐course effect of siPlectin on the phosphorylation of integrin α6β4, activation of FAK and p38. A‐B, Western blot analysis of plectin expression levels after siPlectin transfection. This experiment showed that siRNA #3 (si‐3) was the most efficient siRNA; therefore, this siRNA was used in subsequent experiments. C, Flow cytometry revealed that the apoptosis rate of podocyte was significantly increased after 48 h siPlectin treatment and further increased after 120 and 168 h siPlectin treatment. D, Western blot analysis showed that the protein expression of plectin was significantly decreased after 48 h siPlectin treatment and recovered after 120 h siPlectin treatment. E‐F, Western blot analysis showed that the phosphorylation of integrin α6β4 and FAK peaked at 72 and 120 h, and decreased at 168 h. G, Western blot analysis showed that the phosphorylation of p38 gradually increased after 72 h siPlectin treatment and reached its peak at 168 h

**Figure 5:**
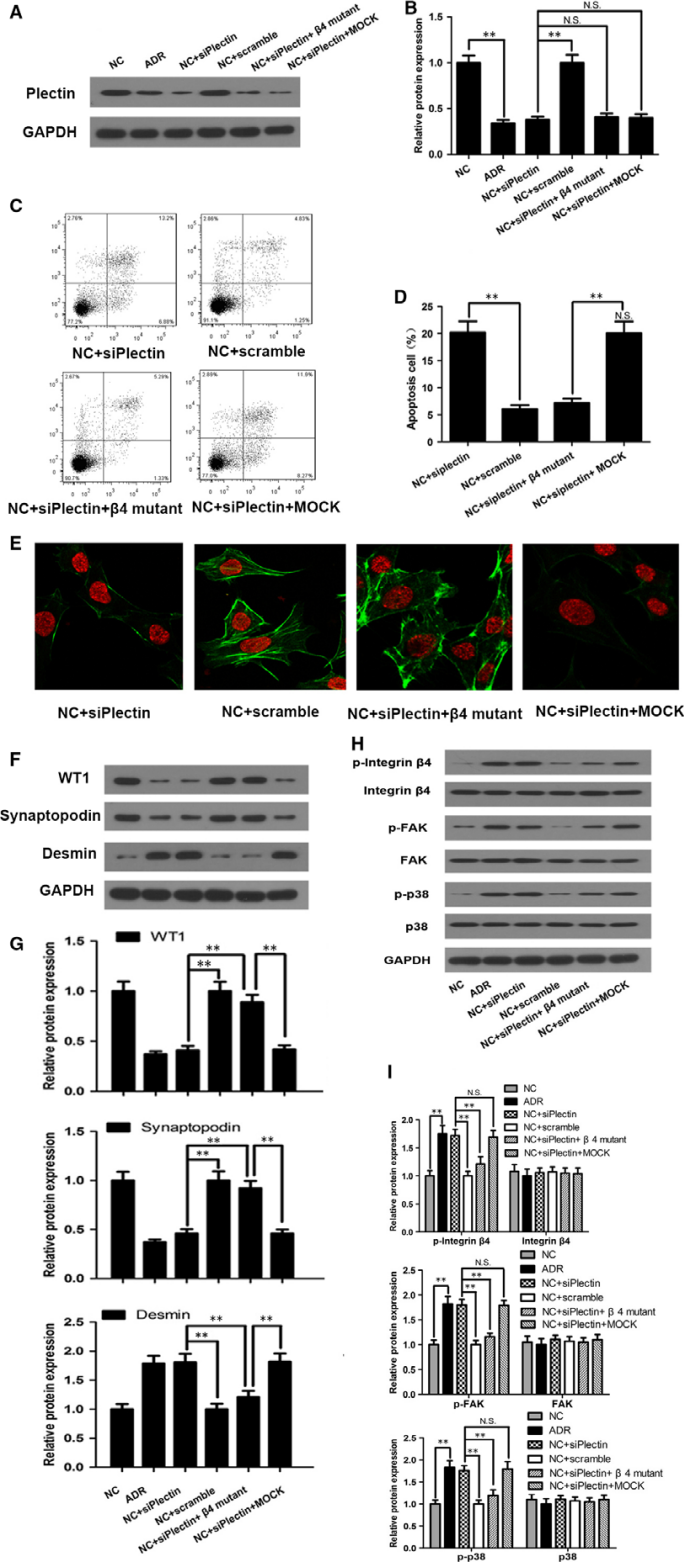
Plectin suppression produced similar effects as ADR‐induced podocyte injury by facilitating integrin α6β4‐mediated FAK and p38 activation. For the ADR group, podocyte was treated with 0.5 μg/mL ADR for 12 h. For the NC + siPlectin group and NC + Scramble group, podocyte was transiently transfected with siPlectin or scrambled RNA at a final concentration of 20 nmol/L for 4 h and then cultured normally for 68 h. A‐B, Western blot showed that plectin protein expression was significantly decreased in the NC + siPlectin group compared with the NC + Scramble groups. Mutating the Y1494 residue of integrin α6β4 in the NC + siPlectin + β4 mutant group had no effect on plectin protein expression. C‐D, Flow cytometry demonstrated increased podocyte apoptosis in the NC + siPlectin group compared with the NC + Scramble group. Mutating integrin α6β4 alleviated siPlectin transfection‐induced apoptosis. E, Immunofluorescence staining demonstrated the presence of significant F‐actin disruption in the NC + siPlectin group compared with the NC + Scramble group. Mutating integrin α6β4 reversed siPlectin transfection‐induced F‐actin disruption. F‐G, Western blot showed that WT1 and synaptopodin expression levels were significantly decreased and that desmin expression levels were significantly increased in the NC + siPlectin group compared with the NC + Scramble group. These abnormalities were partially reversed by integrin α6β4 mutation in NC + siPlectin + β4 mutant group. H‐I, Western blot showed that siPlectin transfection activated integrin α6β4, FAK and p38 phosphorylation, whereas mutating integrin α6β4 abolished the effects of siPlectin. Data shown are representative of three independent experiments (n = 3). **P < 0.01; N.S., not significant. ADR, adriamycin; F‐actin, filamentous actin; FAK, focal adhesion kinase; NC, normal control

To confirm the above findings, we disrupted plectin expression in normal podocytes using siRNA and observed the subsequent changes in podocyte injury. We screened three siRNAs and found that si‐3 most effectively and significantly reduced plectin protein levels in the podocytes (Figure 4A). Western blot analysis showed that plectin levels were significantly decreased in the NC + siPlectin group compared with the NC and NC + scramble groups (Figure 5A,B). Flow cytometry analysis and immunofluorescence staining revealed that suppression of plectin expression caused severe podocyte apoptosis (Figure 5C,D) and F‐actin cytoskeletal disruption (Figure 5E) in the absence of ADR. Furthermore, siPlectin treatment decreased WT1 and synaptopodin expression and increased desmin expression in the siPlectin‐treated group compared with NC and NC + scramble groups (Figure 5F,G). Taken together, these findings indicated that plectin suppression produced similar effects as ADR‐induced podocyte injury.

### 3.5 Plectin suppression led to integrin α6β4, FAK and p38 activation

To elucidate the mechanism by which plectin suppression promotes podocyte injury, we monitored the activity of various signalling pathways in ADR‐ and siPlectin‐treated podocytes. Plectin is known to be an important ligand for integrin α6β4. Phosphorylated integrin α6β4 can recruit and activate FAK,^21^ an important upstream mediator of the p38 pathway.^12^ Both FAK and p38 have been shown to be involved in nephropathy development.^12^ Therefore, we specifically examined integrin α6β4, FAK and p38 expression and activity. Western blot analysis showed that integrin α6β4, FAK and p38 phosphorylation levels were significantly increased in the ADR‐treated group compared with the NC group, whereas total protein levels were similar between the two groups (Figure 3J,K). Recovery of plectin expression significantly abolished ADR‐induced integrin α6β4, FAK and p38 phosphorylation as well as podocyte injury in the ADR + plectin group (Figure 3J,K). In addition, integrin α6β4, FAK and p38 phosphorylation levels were increased in siPlectin‐treated podocytes compared with NC and NC + scramble podocytes, suggesting that plectin suppression alone can induce effects similar those caused by ADR treatment (Figure 5H,I).

### 3.6 The time‐course effect of siPlectin on the phosphorylation of integrin α6β4, activation of FAK and p38

To investigate the time‐course effect of siPlectin on the phosphorylation of integrin α6β4, activation of FAK and p38, as well as induction of cell apoptosis, we collected podocyte at five different time‐points (24, 48, 72, 120 and 168 hours) after siPlectin was added to the podocyte culture medium. We chose these time‐points based on previous studies^22^, ^23^, ^24^, ^25^ in which cells were collected 48 or 72 hours after siPlectin was added to the culture medium and significant down‐regulation of plectin expression and activation of downstream signalling pathways were demonstrated.

Western blot analysis showed that the protein expression of plectin was significantly decreased after 48 hours siPlectin treatment and recovered after 120 hours siPlectin treatment (Figure 4D). The activation of integrin α6β4 and FAK peaked at 72 and 120 hours, and decreased at 168 hours (Figure 4E,F). Activities of p38 gradually increased after 72 hours siPlectin treatment and reached its peak at 168 hours (Figure 4G). Flow cytometry revealed that the apoptosis rate of podocytes was significantly increased after 48 hours siPlectin treatment and further increased after 120 and 168 hours siPlectin treatment (Figure 4C).

### 3.7 Y1494 of integrin α6β4 contributed to plectin‐dependent podocyte injury as well as FAK and p38 activation

The above experiments demonstrated that decreased plectin expression enhanced integrin α6β4, FAK and p38 phosphorylation levels. To investigate the role of integrin α6β4 in podocyte injury and to elucidate the relationship among integrin α6β4, FAK and p38, we blocked Y1494 phosphorylation by transfecting podocytes with vectors containing mutant β4 cDNAs and established stable podocyte subclones expressing mutant Y1494 prior to knocking down plectin with siRNA. We selected residue Y1494 because it is one of the most important phosphorylation sites in the cytoplasmic domain of integrin α6β4. Prior experiments have shown that Y1494 of the β4 subunit is critical for interactions between integrin α6β4 and downstream ligands and that deletion of Y1494 impairs α6β4 signalling.^26^, ^27^ Western blot analysis indicated that Y1494 phosphorylation levels, as well as FAK and p38 phosphorylation levels, were significantly decreased in the NC + siPlectin + β4 mutant group compared with the NC + siPlectin and NC + siPlectin + Mock groups (Figure 5H,I), suggesting that FAK and p38 are downstream effectors of integrin α6β4 whose activation is controlled by plectin. In addition, we found that podocyte injury was mitigated in the NC + siPlectin + β4 mutant group compared to the NC + siPlectin and NC + siPlectin + Mock groups. This change manifested as decreased apoptosis (Figure 5C,D), improved F‐actin cytoskeletal organization (Figure 5E), elevated WT1 and synaptopodin expression, and decreased desmin expression (Figure 5F,G).

### 3.8 Phosphorylation of tyrosine 397 (Y397) on FAK promoted p38 activation

**Figure 6:**
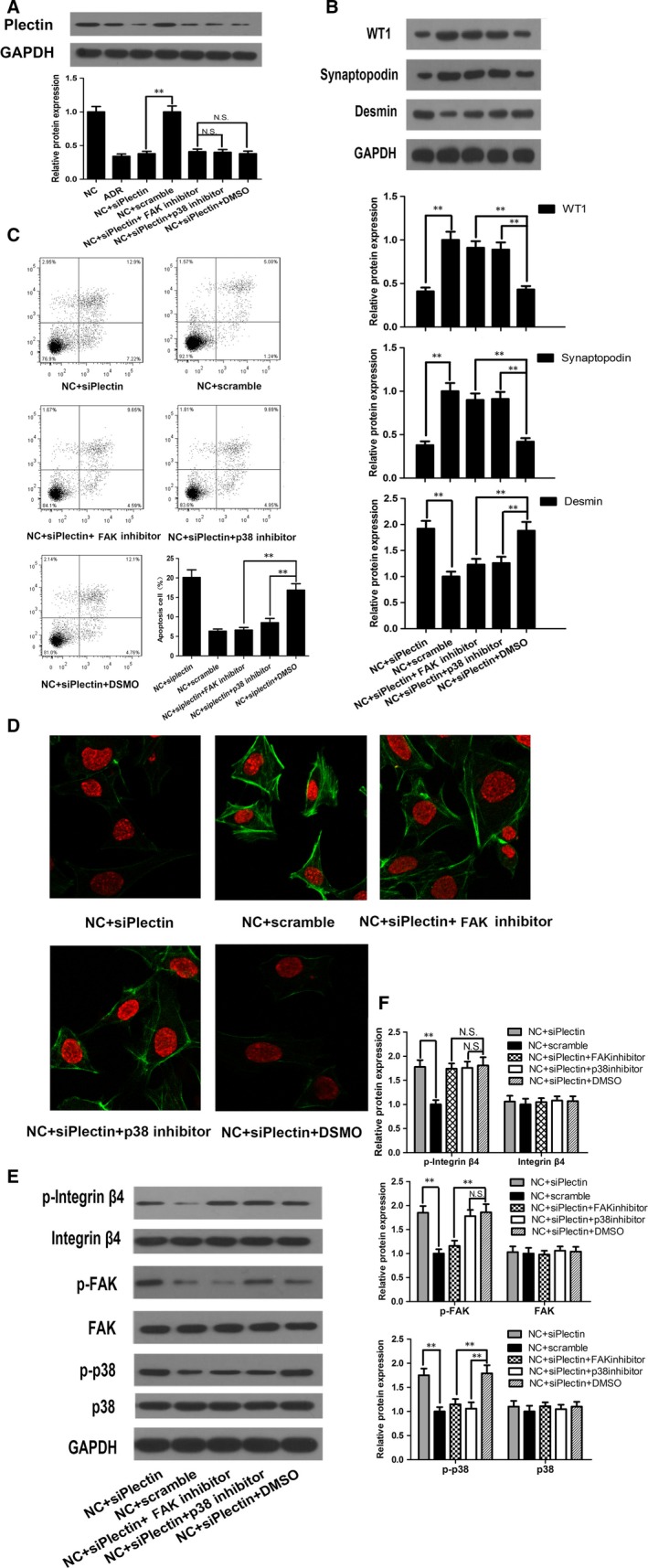
Inhibiting FAK or p38 alleviated siPlectin‐induced podocyte injury. For the NC + siPlectin group and NC + Scramble group, podocyte was transiently transfected with siPlectin or scramble RNA at a final concentration of 20 nmol/L for 4 h and then cultured normally for 68 h. For inhibitor studies, podocyte was preincubated with the FAK inhibitor 14 (50 μmol/L), the p38 inhibitor SB203580 (5 μmol/L) or DMSO vehicle respectively for 1 h before siPlectin transfection and then incubated for an additional 72 h until podocyte collection. A, Western blot showed that siPlectin transfection successfully silenced plectin protein expression in the NC + siPlectin group. Inhibiting FAK in the NC + siPlectin + FAK inhibitor group or p38 in the NC + siPlectin + p38 inhibitor group had no effect on plectin expression. B, Western blot showed that FAK inhibition or p38 inhibition significantly attenuated the abnormalities in WT1, synaptopodin and desmin expression induced by siPlectin. C, Flow cytometry showed that FAK inhibition or p38 inhibition significantly alleviated siPlectin‐induced apoptosis. D, Immunofluorescence staining showed that FAK inhibition or p38 inhibition reversed siPlectin‐induced F‐actin disruption. E‐F, Western blot showed that integrin α6β4, FAK and p38 phosphorylation levels were elevated in the NC + siPlectin group. FAK inhibition at the Y397 site did not affect siPlectin‐induced integrin α6β4 phosphorylation levels but decreased p38 phosphorylation levels in the NC + siPlectin + FAK inhibitor group compared with the NC + siPlectin + DMSO group. p38 inhibition in the NC + siPlectin + p38 inhibitor group had no impact on integrin α6β4 and FAK phosphorylation levels. Data shown are representative of three independent experiments (n = 3). **P < 0.01; N.S., not significant. ADR, adriamycin; F‐actin, filamentous actin; FAK, focal adhesion kinase; NC, normal control

Activated integrin α6β4 has been shown to recruit FAK, thereby inducing Y397 autophosphorylation^21^ and triggering the expression of downstream effectors, such as p38.^12^ Thus, we evaluated the effects of phosphorylated FAK Y397 on podocyte injury and the relationship between FAK and p38. Western blot analysis showed that FAK inhibitor 14, a specific FAK Y397 inhibitor,^21^ suppressed p38 activation by preventing FAK Y397 phosphorylation but had no effect on integrin α6β4 phosphorylation, suggesting that FAK is an upstream inducer of p38 and a downstream effector of integrin α6β4 (Figure 6E,F). Western blot analysis also showed that WT1 and synaptopodin expression levels were higher, and desmin expression levels were lower in the NC + siPlectin + FAK inhibitor 14 group than in the NC + siPlectin and DMSO groups (Figure 6B). Flow cytometry analysis and immunofluorescence staining also showed that podocyte injury was attenuated in the NC + siPlectin + FAK inhibitor 14 group compared with the NC + siPlectin and DMSO groups. These changes manifested as decreases in apoptosis (Figure 6C) and improvements in F‐actin cytoskeletal reorganization (Figure 6D). Our findings suggested that blocking FAK Y397 phosphorylation inhibited p38 activation, thereby attenuating podocyte injury.

### 3.9 p38 activation triggered apoptosis and F‐actin disruption

**Figure 7:**
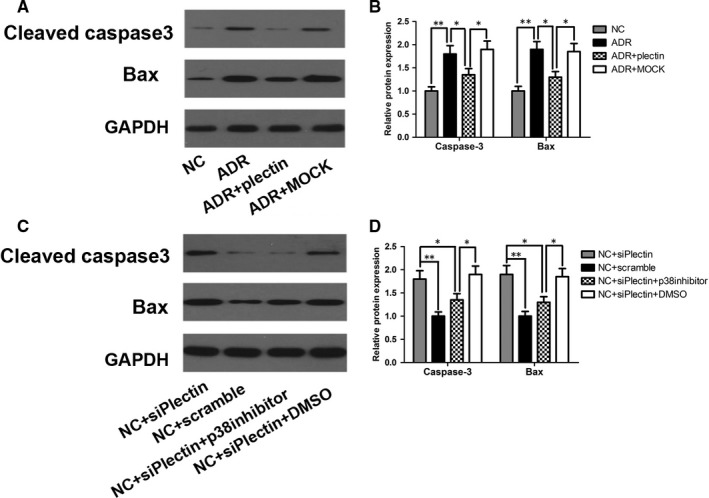
p38 induced podocyte apoptosis by activating Bax and caspase‐3. For the ADR group, podocyte was treated with 0.5 μg/mL ADR for 12 h. For the ADR + plectin group or ADR + MOCK group, podocyte was incubated with pEGFP‐N1‐plectin plasmids or empty vectors for 6 h and then cultured normally for 42 h, 0.5 μg/mL ADR was added 12 h prior to cell harvest. For the NC + siPlectin group and NC + Scramble group, podocyte was transfected with siPlectin or scramble RNA respectively at a final concentration of 20 nmol/L for 4 h and then cultured normally for 68 h. For p38‐MAPK inhibitor study, podocyte was preincubated with the p38 inhibitor SB203580 (5 μmol/L) or DMSO vehicle for 1 h before siPlectin transfection and then incubated for an additional 72 h until podocyte collection. A‐B, Western blot showed that Bax and cleaved caspase‐3 protein expression levels were elevated in ADR‐treated podocyte compared with NC podocyte and that recovering plectin expression inhibited the activation of these pro‐apoptotic proteins. C‐D, Western blot showed that siPlectin transfection increased Bax and cleaved caspase‐3 protein expression in the NC + siPlectin group compared with the NC + Scramble group, whereas p38 inhibition blocked the siPlectin‐induced activation of these proteins. Data shown are representative of three independent experiments (n = 3). *P < 0.05 and **P < 0.01

p38 is believed to be a critical mediator of cell apoptosis^28^ and a regulator of cytoskeletal stability.^4^ p38 also plays a key role in the progression of podocyte injury and proteinuria in various glomerulopathies.^29^ These findings prompted us to investigate the role of p38 in podocyte injury. We demonstrated that treatment with SB203580, a p38 inhibitor, significantly abolished siPlectin‐stimulated p38 activation (Figure 6E,F) and attenuated podocyte injury (Figure 6B‐D) in the NC + siPlectin + p38 inhibitor group compared with the NC + siPlectin group. These findings suggest that p38 activation is a common upstream mechanism that plays an essential role in podocyte injury in proteinuric glomerulopathies. Western blot analysis showed that SB203580 treatment suppressed p38 phosphorylation without affecting the increases in β4 integrin and FAK phosphorylation (Figure 6E,F), indicating that p38 may serve as a downstream effector of integrin α6β4 and FAK in response to plectin suppression. Bax and cleaved caspase‐3 are essential mediators of apoptosis. Synaptopodin is an actin‐associated adapter that plays important roles in podocyte F‐actin reorganization and, consequently, proteinuria.^30^ In this study, siPlectin treatment increased Bax and cleaved caspase‐3 expression and decreased synaptopodin expression (Figure 7C,D). These changes were reversed by SB203580, suggesting that phosphorylated p38 triggers apoptosis and F‐actin cytoskeletal disruption by up‐regulating Bax and cleaved caspase‐3 expression and down‐regulating synaptopodin expression, respectively.

### 3.10 ADR treatment decreased plectin expression, activated integrin α6β4, FAK and p38, and induced renal injury in rats

The mean body weights of the two groups were similar before ADR treatment. Four weeks later, the body weight of the ADR‐treated group was significantly lower than that of the NC group. The 24‐hour UP, BUN and SCr levels were significantly higher in the ADR group than in the NC group, and 24‐hour UV was significantly lower in the ADR group than in the NC group. These results are shown in Table 2.

**Table 2:** 24‐h UP, 24‐h UV, BUN, SCr and weight in the two groups

Group	n	24 h UV (mL)	24 h UP (mg)	BUN (mmol/L)	SCr (μmol/L)	Weight (mg)
NC	10	25 ± 3.98	3.47 ± 0.55	7.59 ± 0.93	45.32 ± 4.66	226 ± 18
ADR	10	6 ± 1.27^a^	27.17 ± 2.51^a^	18.36 ± 2.98^a^	117.03 ± 14.16^a^	148 ± 14^a^

24‐h UP, 24‐h urine protein; 24‐h UV, 24‐h urine volume; ADR, Adriamycin; BUN, blood urea nitrogen; NC, normal control; SCr, serum creatinine.

Values are expressed as the mean ± SD.

a *P* < 0.01 vs NC group.

**Figure 8:**
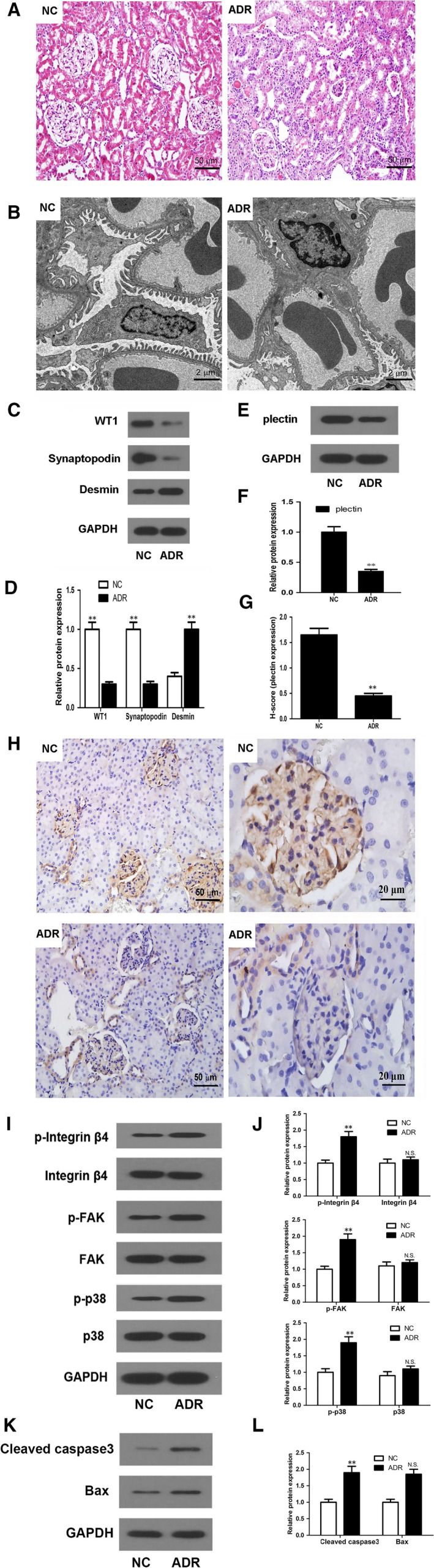
ADR contributed to the development of proteinuria and renal dysfunction by inhibiting plectin expression and activating the integrin α6β4/FAK/p38 pathway. ADR induced nephropathy was introduced by a single injection of 7.5 mg/kg ADR via the tail vein. All rats were killed at the end of the 4th week after ADR injection. A, Light microscopic examination revealed glomerular atrophy and disappearance as well as renal tubular swelling after ADR treatment. B, TEM examination of the NC group revealed the presence of normal glomerular ultrastructure. TEM examination of the ADR group revealed the presence of diffuse foot process effacement, GBM thickening, slit diaphragm loss and mesangial sclerosis. C‐D, Western blot showed that WT1 and synaptopodin protein expression levels were decreased and that desmin protein expression levels were increased in the ADR group compared with the NC group. E, Western blot showed that plectin expression was suppressed in the ADR‐treated group (n = 5) compared with the NC group (n = 5). F‐G, Immunohistochemical analysis showed that plectin expression in glomeruli was decreased in ADR treated kidney tissues (n = 5) compared with normal kidney tissues (n = 5). H‐I, Western blot showed that integrin α6β4, FAK and p38 were activated by ADR treatment. J‐K, Western blot showed that cleaved caspase‐3 and Bax protein expression was increased in the ADR group (n = 5) compared with the NC group (n = 5). **P < 0.01

Light microscopic examination revealed the presence of significant histopathologic changes in the glomerular and tubulo‐interstitial areas of the rat kidneys in the ADR group compared with those in the NC group (Figure 8A). The glomerular changes included glomerular atrophy and loss, Bowman space expansion, glomerular mesangial cell proliferation and increased mesangial matrix deposition, and the tubular changes included tubular expansion, tubular epithelial cell swelling, inflammatory cell infiltration in the renal interstitium, and proteinaceous cast formation.

Podocyte foot process effacement is an early finding in many glomerular diseases and accompanies podocyte slit diaphragm loss and proteinuria development.^31^ Electron microscopy revealed the presence of preserved thin and long foot processes in the NC group; however, foot process diffusion and effacement as well as glomerular basement membrane (GBM) thickening, slit diaphragm loss and mesangial sclerosis were observed in the ADR‐treated group at 4 weeks after ADR injection (Figure 8B).

The protein levels of the classic podocyte markers WT1 and synaptopodin as well as those of the injury marker desmin were also detected in vivo using Western blot. WT1 and synaptopodin expression levels were significantly decreased while desmin expression levels were increased in ADR‐treated rats compared with NC rats (Figure 8C,D).

To determine whether plectin played a role in renal injury in vivo, we examined plectin protein expression in the renal cortices of the rats. Western blot analysis showed that plectin expression levels were significantly decreased in the ADR group compared with the NC group (Figure 8E,F). Plectin down‐regulation in the podocytes of ADR‐treated rats was confirmed by immunohistochemical staining of the rat renal tissues (Figure 8G,H). A previous study verified that plectin is predominantly localized in the perinuclear regions of podocytes^32^; however, we found that plectin was not only localized in the podocytes of the glomeruli but also expressed in the epithelial cells of the collecting ducts (Figure 6H). Consistent with the Western blot analysis results, the findings from the immunohistochemical staining experiments showed that the plectin staining intensity in podocytes was significantly decreased in the ADR group compared with the NC group (Figure 6G). However, the staining intensity in the epithelial cells of the collecting ducts was not significantly different between the two groups (data not shown).

Integrin α6β4, FAK and p38 expression and activity were examined in vivo. Western blot assays showed that integrin α6β4, FAK and p38 phosphorylation levels were significantly increased in the ADR‐treated group compared with the NC group, whereas the total protein levels were similar between the two groups (Figure 8I,J). Moreover, the expression levels of the podocyte apoptosis markers Bax and cleaved caspase‐3 were significantly up‐regulated in the ADR‐treated group compared with the NC group (Figure 8K,L).

## 4 DISCUSSION

**Figure 9:**
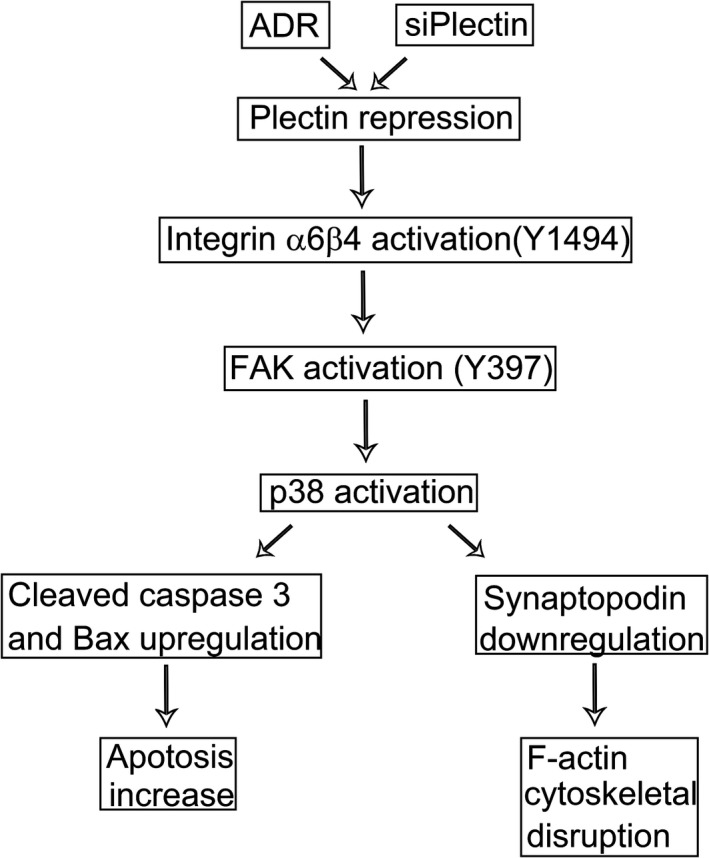
Schematic representation of the mechanism by which plectin repression promotes apoptosis and F‐actin cytoskeletal disruption in podocyte

Podocytes are an important component of the glomerular membrane. Precise podocyte actin cytoskeletal organization and regulation are essential for maintaining an intact glomerular filtration barrier and for acclimatizing podocytes to environmental changes. Disrupting the actin cytoskeleton of podocytes induces changes in their shape, motility and adhesion, resulting in foot process effacement and proteinuria in glomerular diseases. Apoptosis is another important consequence of podocyte injury. Podocyte numbers and integrity are critical for the maintenance of normal glomerular filtration, and apoptosis‐induced decreases in podocyte numbers are crucial events in the pathogenesis of proteinuria following podocyte injury. Therefore, actin cytoskeletal regulation and apoptosis regulation in podocytes are both believed to be vital targets for the prevention of proteinuria development in various forms of chronic kidney disease (Figure 9).

Our study demonstrated that plectin, a cytoskeleton linker protein, had a substantial impact on F‐actin cytoskeletal assembly and podocyte apoptosis and thus plays an important role in podocyte function. Plectin, a 500‐kD multidomain protein extensively expressed in podocytes, has a dumbbell‐like structure comprising a central 200‐nm‐long rod domain flanked by two large globular domains.^33^ This multidomain structure enables plectin to interact with a vast array of different protein and significantly affect their functionality.^34^ In this study, we adopted the widely used ADR‐induced podocyte injury model. This model is ideal for elucidating the mechanisms underlying podocyte injury, as it is characterized by F‐actin cytoskeletal damage and podocyte apoptosis and simulates the changes in podocyte structure and viability that are characteristic of glomerular diseases. We consistently observed severe F‐actin cytoskeleton disaggregation in ADR‐treated podocytes, which displayed decreased actin content and disrupted actin stress fibres. Interestingly, remarkably decreased plectin expression was observed in ADR‐treated podocytes compared with NC podocytes. Restoration of plectin expression via transfection of podocytes with plasmids containing plectin cDNA protected against ADR‐induced podocyte injury. Moreover, siRNA‐mediated plectin silencing induced similar effects as ADR‐induced podocyte injury, including increased apoptosis and aggravated F‐actin cytoskeletal disarrangement compared with NC cells. Furthermore, the results of the in vivo study involving the ADR rat model also suggested that decreases in plectin expression were related to podocyte injury and the subsequent development of proteinuria.

To elucidate the mechanism by which plectin is trafficked in podocyte, we focused on the function of integrins family of podocyte. Integrins are heterodimeric membrane‐spanning adhesion receptors that are essential for a wide range of biological functions. Integrin activation is controlled by competitive interactions involving the cytoplasmic domains, particularly the β‐tails.^35^ Previous studies about podocyte injury mainly focused on integrin β1 or integrin β3. For example, NF‐kappa B and other pro‐inflammatory transcription factors can induce the activation of downstream integrin β3, leading to podocyte injury and proteinuria.^36^ Whereas the expression of integrin β1 in podocyte was considered negatively correlated with the severity of proteinuria.^37^ Elevated expression of TGF‐β1 or ROS induced decreased expression of integrin β1, resulting in the production of proteinuria.^38^, ^39^ In contrast to the other β subunits, the integrin β4 subunit contains an exceptionally large cytoplasmic domain comprising several major tyrosine phosphorylation sites and thus regulates multiple intracellular signalling pathways.^40^, ^41^ The integrin β4 subunit can pair only with the integrin α6 subunit^42^; thus, the expression levels of the β4 subunit can be used to reflect the expression levels of integrin α6β4. Few studies on integrin α6β4 in podocytes have been conducted. The majority of previous studies on integrin α6β4 have focused on the hemidesmosome in various epithelial cells. Integrin α6β4‐plectin binding is essential for the formation and stabilization of the hemidesmosome, an anchoring structure that mediates the attachment of epithelial cells to the cell substratum.^9^ Podocytes are specialized epithelial cells whose foot processes attach to the GBM. The F‐actin‐based cytoskeleton of podocytes stabilizes these foot processes and is linked to adhesion receptors, such as integrins, through focal contacts, which allow podocytes to form strong adhesive bonds with the GBM.^17^ As the foot processes of podocytes play a role similar to that of hemidesmosomes in epithelial cells, it is not difficult to imagine that the interactions between integrin α6β4 and plectin are also important for podocyte structure and function.

There are mainly two important combinations between plectin and integrin β4. The first interaction occurs between the actin‐binding domain of plectin and the first pair of fibronectin type III (FNIII‐1,2) domains and a small part of the connecting segment (CS) of β4.^8^, ^43^, ^44^ The second interaction occurs between the plakin domain of plectin and the CS and C‐tail of integrin β4.^45^, ^46^ When plectin binds to integrin β4, FNIII‐3, FNIII‐4 and the C‐terminal tail of β4 are crimped into a loop structure.^9^, ^47^ Y1494 is a critical tyrosine residue localized in the FNIII‐3 domain of the integrin β4.^48^ The results in this study showed that the Y1494 site of integrin β4 was phosphorylated when plectin expression was down‐regulated. According to the molecular spatial conformation that plectin binds to integrin β4, we speculate that, when plectin is bound to integrin β4, the Y1494 site in the FNIII‐3 domain of integrin β4 is covered in the loop structure and cannot be phosphorylated. When plectin is down‐regulated and dissociates from integrin β4, the spatial conformation of the C‐terminus of β4 changes, leaving the loop structure open to a linear structure.^49^, ^50^ At this time, Y1494 residue at the FNIII‐3 domain of β4 is exposed and can be phosphorylated. Thus, it is reasonable to speculate that plectin‐integrin α6β4 binding may maintain Y1494 in an inhibited state and that loss of plectin‐integrin α6β4 binding results in the dissociation of Y1494 from integrin α6β4 and subsequent phosphorylation of integrin α6β4 by various upstream factors. In this study, we noted that suppression of plectin expression directly elevated Y1494 phosphorylation in ADR‐treated podocytes and plectin‐knockdown podocytes compared with NC podocyte; these findings support the above hypothesis. Previous studies have shown that integrin α6β4‐plectin binding was dynamically regulated by integrin α6β4 phosphorylation.^51^ Specifically, β4 phosphorylation prevented plectin from binding with integrin α6β4. The results of our study showed for the first time that the binding of plectin to integrin α6β4 also conversely inhibits integrin α6β4 phosphorylation. We noted that total integrin α6β4 levels were mildly decreased in ADR‐treated podocyte compared with NC podocyte. However, in contrast to phosphorylated integrin α6β4 levels, total integrin α6β4 levels were not affected by plectin expression, indicating that integrin α6β4 phosphorylation rather than total integrin α6β4 expression induces the abovementioned changes in podocyte.

Phosphorylated integrin β4 successively activates FAK and p38, a phenomenon that may represent a core mechanism underlying F‐actin cytoskeletal rearrangement and podocyte apoptosis. We mutated Y1494 of the integrin β4 subunit and used FAK inhibitor 14 and the p38 inhibitor SB203580 to confirm the upstream and downstream relationships among the above molecules. The results showed that mutating Y1494 of integrin β4 decreased FAK and p38 phosphorylation as well as attenuated apoptosis and F‐actin cytoskeletal rearrangement, suggesting that integrin β4 is the upstream inducer of FAK and p38. We also found that FAK inhibitor 14 inhibited FAK and p38 activity but had no impact on integrin β4 phosphorylation levels, suggesting that FAK is the upstream inducer of p38. Meanwhile, SB203580 prevented p38‐induced apoptosis and F‐actin reorganization but failed to alter integrin β4 and FAK activation, indicating that p38 is a downstream effector of FAK signalling and may also be a direct inducer of apoptosis and F‐actin cytoskeletal rearrangement.

Focal adhesion kinase is an intracellular non‐receptor tyrosine kinase and a classical downstream target of integrins. Multiple studies have provided evidence indicating that FAK is expressed in podocytes and is activated in many podocyte pathologies and glomerular diseases.^4^, ^14^ A previous study showed that integrin α5β3‐mediated FAK/PI3K pathway activation played a critical role in podocyte F‐actin cytoskeletal rearrangement.^52^ Our study demonstrated that integrin α6β4, another integrin family heterodimer, also activated FAK and induced actin filament rearrangements. Consistent with the results of our investigation, the results of studies involving other cell lines have shown that activated integrin α6β4 can recruit FAK, thereby inducing autophosphorylation at Y397,^21^ a critical tyrosine site that plays a role in the activation of downstream effectors, such as p38.P38 has been demonstrated to mediate podocyte injury in various models of renal disease; moreover, p38 inhibition has been shown to protect podocytes from injury in vitro and ameliorate proteinuria in vivo.^29^, ^53^ A recent study showed that the FAK/p38 axis contributed to foot process effacement and proteinuria development in a rat model of short‐term hypercholesterolaemia by modulating podocyte apoptosis and F‐actin reorganization.^4^ Consistent with this finding, the results of our study demonstrated that the FAK/p38 axis plays an important role in ADR‐induced podocyte injury and showed that plectin and integrin α6ß4 are the upstream inducers of this axis.

P38 likely mediates podocyte injury by triggering apoptosis and F‐actin reorganization. p38 activation has been shown in many previous studies to be strongly correlated with increases in the expression levels of the apoptosis‐related proteins Bax and cleaved caspase‐3.^54^, ^55^ In this study, p38 inhibition reduced siPlectin‐induced podocyte apoptosis and decreased Bax and cleaved caspase‐3 expression, findings that are consistent with those of prior studies. Previous studies have also shown that p38 activation inhibits actin polymerization and decreases F‐actin levels to maintain a dynamic and balanced F‐actin cytoskeleton and normal podocyte foot process structures.^4^ These findings were also observed in siPlectin‐treated podocyte and ADR‐treated rats in this study. Synaptopodin is an important slit diaphragm protein and an actin‐organizing protein that shows a fibre‐like pattern characteristic of peripheral actin stress fibres. Synaptopodin protects the podocyte cytoskeleton in vitro and safeguards against filter barrier damage in mice by dynamically controlling the actin cytoskeleton in podocytes.^35^, ^56^ Synaptopodin down‐regulation drives major actin cytoskeletal reorganization, foot process effacement and proteinuria development.^57^, ^58^ In this study, F‐actin cytoskeletal disaggregation in siPlectin‐treated podocyte occurred in conjunction with p38 activation and synaptopodin down‐regulation. Pretreating siPlectin‐treated podocytes with a p38 inhibitor restored actin stress fibre integrity and synaptopodin expression to a pattern comparable with that observed in normal podocytes. The above results indicated that p38 likely mediates F‐actin reorganization by controlling synaptopodin expression.

The in vivo study can only display that plectin and integrin α6β4/FAK/p38 MAPK pathway are involved in ADR‐induced foot process effacement and proteinuria, but cannot further elaborate the relationship between them because the plectin expression in podocytes was not restored successfully in vivo. We tried to restore the expression of plectin in the glomeruli (especially podocytes) of ADR‐treated kidney tissues by injecting recombinant adenovirus vector encoding plectin (Plectin‐Ad‐EGFP) via tail vein. EGFP expression in the kidney tissues was observed with a fluorescence microscope (DX51; Olympus) to determine transfection efficiency. The results showed that EGFP was predominantly expressed in renal tubular epithelial cells with little expression in glomeruli. Western blot assay was also performed and the results showed that the protein expression of plectin in glomeruli of rats injected with Plectin‐Ad‐EGFP had no significant difference with that of the control rats. We adjusted the injection dose of Plectin‐Ad‐EGFP and the time‐point of organ harvest, but the results remained the same (data not shown here). These results suggest that adenovirus did not successfully enter the glomeruli, but selectively transfected into renal tubular epithelial cells. Therefore, the limitation for this part is the lack of the evidence showing in vivo function of plectin in protecting podocytes and regulating the downstream signalling pathways.

## 5 CONCLUSION

In summary, our in vitro study demonstrated that ADR‐ or siRNA‐induced decreases in the expression of the cytoskeletal protein plectin activated integrin α6β4, FAK and p38 and ultimately induced podocyte apoptosis and F‐actin reorganization. The in vivo study showed that plectin and integrin α6β4/FAK/p38 pathway are involved in ADR‐induced foot process effacement and proteinuria. This study has identified novel targets that may be used for the development of therapies for podocyte injury‐related glomerulopathies.

## CONFLICT OF INTEREST

All the co‐authors declare that we have no conflict of interests in the submission of this manuscript.

## References

[1] Tagawa A , Yasuda M , Kume S , et al. Impaired podocyte autophagy exacerbates proteinuria in diabetic nephropathy. Diabetes. 2016;65:755‐767. 26384385 10.2337/db15-0473

[2] Schwartzman M , Reginensi A , Wong JS , et al. Podocyte‐specific deletion of yes‐associated protein causes FSGS and progressive renal failure. J Am Soc Nephrol. 2016;27:216‐226. 26015453 10.1681/ASN.2014090916 PMC4696566

[3] Allison SJ . Chronic kidney disease: actin cytoskeleton alterations in podocytes: a therapeutic target for chronic kidney disease. Nat Rev Nephrol. 2015;11:385. 25963588 10.1038/nrneph.2015.79

[4] Hu M , Fan M , Zhen J , et al. FAK contributes to proteinuria in hypercholesterolaemic rats and modulates podocyte F‐actin re‐organization via activating p38 in response to ox‐LDL. J Cell Mol Med. 2017;21:552‐567. 27704688 10.1111/jcmm.13001 PMC5323874

[5] Schiffer M , Teng B , Gu C , et al. Pharmacological targeting of actin‐dependent dynamin oligomerization ameliorates chronic kidney disease in diverse animal models. Nat Med. 2015;21:601‐609. 25962121 10.1038/nm.3843 PMC4458177

[6] Jiu Y , Lehtimaki J , Tojkander S , et al. Bidirectional interplay between vimentin intermediate filaments and contractile actin stress fibers. Cell Rep. 2015;11:1511‐1518. 26027931 10.1016/j.celrep.2015.05.008

[7] Bouameur JE , Favre B , Borradori L . Plakins, a versatile family of cytolinkers: roles in skin integrity and in human diseases. J Invest Dermatol. 2014;134:885‐894. 24352042 10.1038/jid.2013.498

[8] de Pereda JM , Lillo MP , Sonnenberg A . Structural basis of the interaction between integrin alpha6beta4 and plectin at the hemidesmosomes. EMBO J. 2009;28:1180‐1190. 19242489 10.1038/emboj.2009.48 PMC2683700

[9] Song JG , Kostan J , Drepper F , et al. Structural insights into Ca^2+^‐calmodulin regulation of Plectin 1a‐integrin beta4 interaction in hemidesmosomes. Structure. 2015;23:558‐570. 25703379 10.1016/j.str.2015.01.011 PMC4353693

[10] Gregor M , Osmanagic‐Myers S , Burgstaller G , et al. Mechanosensing through focal adhesion‐anchored intermediate filaments. FASEB J. 2014;28:715‐729. 24347609 10.1096/fj.13-231829

[11] Cheng CC , Lai YC , Lai YS , et al. Transient knockdown‐mediated deficiency in plectin alters hepatocellular motility in association with activated FAK and Rac1‐GTPase. Cancer Cell Int. 2015;15:29. 25774093 10.1186/s12935-015-0177-1 PMC4358909

[12] Alanko J , Ivaska J . Endosomes: emerging platforms for integrin‐mediated FAK signalling. Trends Cell Biol. 2016;26:391‐398. 26944773 10.1016/j.tcb.2016.02.001

[13] Nader GP , Ezratty EJ , Gundersen GG . FAK, talin and PIPKIgamma regulate endocytosed integrin activation to polarize focal adhesion assembly. Nat Cell Biol. 2016;18:491‐503. 27043085 10.1038/ncb3333

[14] Ma H , Togawa A , Soda K , et al. Inhibition of podocyte FAK protects against proteinuria and foot process effacement. J Am Soc Nephrol. 2010;21:1145‐1156. 20522532 10.1681/ASN.2009090991 PMC3152231

[15] Zhao SH , Gao HQ , Ji X , et al. Effect of ouabain on myocardial ultrastructure and cytoskeleton during the development of ventricular hypertrophy. Heart Vessels. 2013;28:101‐113. 22241736 10.1007/s00380-011-0219-0

[16] Zhao SH , Qiu J , Wang Y , et al. Profilin‐1 promotes the development of hypertension‐induced cardiac hypertrophy. J Hypertens. 2013;31:576‐586; discussion 86. 23615214 10.1097/HJH.0b013e32835d6a56

[17] Reiser J , Sever S . Podocyte biology and pathogenesis of kidney disease. Annu Rev Med. 2013;64:357‐366. 23190150 10.1146/annurev-med-050311-163340 PMC3736800

[18] Wegener KL , Campbell ID . Transmembrane and cytoplasmic domains in integrin activation and protein‐protein interactions (review). Mol Membr Biol. 2008;25:376‐387. 18654929 10.1080/09687680802269886 PMC3000922

[19] Daehn I , Casalena G , Zhang T , et al. Endothelial mitochondrial oxidative stress determines podocyte depletion in segmental glomerulosclerosis. J Clin Investig. 2014;124:1608‐1621. 24590287 10.1172/JCI71195 PMC3973074

[20] Papeta N , Zheng Z , Schon EA , et al. Prkdc participates in mitochondrial genome maintenance and prevents Adriamycin‐induced nephropathy in mice. J Clin Investig. 2010;120:4055‐4064. 20978358 10.1172/JCI43721 PMC2964992

[21] Lakshmanan I , Rachagani S , Hauke R , et al. MUC5AC interactions with integrin beta4 enhances the migration of lung cancer cells through FAK signaling. Oncogene. 2016;35:4112‐4121. 26751774 10.1038/onc.2015.478 PMC5745007

[22] Niwa T , Saito H , Imajoh‐ohmi S , et al. BRCA2 interacts with the cytoskeletal linker protein plectin to form a complex controlling centrosome localization. Cancer Sci. 2009;100:2115‐2125. 19709076 10.1111/j.1349-7006.2009.01282.x PMC11158164

[23] Cheng CC , Lai YC , Lai YS , et al. Cell pleomorphism and cytoskeleton disorganization in human liver cancer. In Vivo. 2016;30:549‐555. 27566071

[24] Katada K , Tomonaga T , Satoh M , et al. Plectin promotes migration and invasion of cancer cells and is a novel prognostic marker for head and neck squamous cell carcinoma. J Proteomics. 2012;75:1803‐1815. 22245045 10.1016/j.jprot.2011.12.018

[25] Liu YH , Cheng CC , Lai YS , et al. Synemin down‐regulation in human hepatocellular carcinoma does not destabilize cytoskeletons in vivo. Biochem Biophys Res Comm. 2011;404:488‐493. 21144834 10.1016/j.bbrc.2010.12.008

[26] Dutta U , Shaw LM . A key tyrosine (Y1494) in the beta4 integrin regulates multiple signaling pathways important for tumor development and progression. Can Res. 2008;68:8779‐8787. 10.1158/0008-5472.CAN-08-2125 PMC2586898 18974120

[27] Ge D , Kong X , Liu W , et al. Phosphorylation and nuclear translocation of integrin beta4 induced by a chemical small molecule contribute to apoptosis in vascular endothelial cells. Apoptosis. 2013;18:1120‐1131. 23677256 10.1007/s10495-013-0860-4

[28] Liu WT , Peng FF , Li HY , et al. Metadherin facilitates podocyte apoptosis in diabetic nephropathy. Cell Death Dis. 2016;7:e2477. 27882943 10.1038/cddis.2016.335 PMC5260885

[29] Koshikawa M , Mukoyama M , Mori K , et al. Role of p38 mitogen‐activated protein kinase activation in podocyte injury and proteinuria in experimental nephrotic syndrome. J Am Soc Nephrol. 2005;16:2690‐2701. 15987752 10.1681/ASN.2004121084

[30] Yaddanapudi S , Altintas MM , Kistler AD , et al. CD2AP in mouse and human podocytes controls a proteolytic program that regulates cytoskeletal structure and cellular survival. J Clin Investig. 2011;121:3965‐3980. 21911934 10.1172/JCI58552 PMC3195478

[31] Gee HY , Zhang F , Ashraf S , et al. KANK deficiency leads to podocyte dysfunction and nephrotic syndrome. J Clin Investig. 2015;125:2375‐2384. 25961457 10.1172/JCI79504 PMC4497755

[32] Yaoita E , Wiche G , Yamamoto T , Kawasaki K , Kihara I . Perinuclear distribution of plectin characterizes visceral epithelial cells of rat glomeruli. Am J Pathol. 1996;149:319‐327. 8686756 PMC1865232

[33] Steinbock FA , Nikolic B , Coulombe PA , Fuchs E , Traub P , Wiche G . Dose‐dependent linkage, assembly inhibition and disassembly of vimentin and cytokeratin 5/14 filaments through plectin's intermediate filament‐binding domain. J Cell Sci. 2000;113(Pt 3):483‐491. 10639335 10.1242/jcs.113.3.483

[34] Winter L , Staszewska I , Mihailovska E , et al. Chemical chaperone ameliorates pathological protein aggregation in plectin‐deficient muscle. J Clin Investig. 2014;124:1144‐1157. 24487589 10.1172/JCI71919 PMC3934181

[35] Faul C , Donnelly M , Merscher‐Gomez S , et al. The actin cytoskeleton of kidney podocytes is a direct target of the antiproteinuric effect of cyclosporine A. Nat Med. 2008;14:931‐938. 18724379 10.1038/nm.1857 PMC4109287

[36] Prakoura N , Kavvadas P , Kormann R , Dussaule JC , Chadjichristos CE , Chatziantoniou C . NFkappaB‐induced periostin activates integrin‐beta3 signaling to promote renal injury in GN. J Am Soc Nephrol. 2017;28:1475‐1490. 27920156 10.1681/ASN.2016070709 PMC5407726

[37] Cheng YC , Chen CA , Chang JM , Chen HC . Albumin overload down‐regulates integrin‐beta1 through reactive oxygen species‐endoplasmic reticulum stress pathway in podocytes. J Biochem. 2015;158:101‐108. 25713411 10.1093/jb/mvv020

[38] Liu J , Zhang B , Chai Y , Xu Y , Xing C , Wang X . Fluvastatin attenuated the effect of expression of beta1 integrin in PAN‐treated podocytes by inhibiting reactive oxygen species. Mol Cell Biochem. 2015;398:207‐215. 25240415 10.1007/s11010-014-2220-2

[39] Zhu Y , Shen J , Hu YY , Tang JL , Liu S . [Transforming growth factor‐beta1 regulates renal alpha3 and beta1 integrin expressions in diabetic rats: a new insight into the renoprotective effect of irbesartan]. Nan Fang Yi Ke Da Xue Xue Bao. 2011;31:1059‐1062. 21690069

[40] Chapman HA , Li X , Alexander JP , et al. Integrin alpha6beta4 identifies an adult distal lung epithelial population with regenerative potential in mice. J Clin Investig. 2011;121:2855‐2862. 21701069 10.1172/JCI57673 PMC3223845

[41] Zhou X , Matskova L , Rathje LS , et al. SYK interaction with ITGbeta4 suppressed by Epstein‐Barr virus LMP2A modulates migration and invasion of nasopharyngeal carcinoma cells. Oncogene. 2015;34:4491‐4499. 25531330 10.1038/onc.2014.380

[42] Stewart RL , O'Connor KL . Clinical significance of the integrin alpha6beta4 in human malignancies. Lab Invest. 2015;95:976‐986. 26121317 10.1038/labinvest.2015.82 PMC4554527

[43] Rezniczek GA , de Pereda JM , Reipert S , Wiche G . Linking integrin alpha6beta4‐based cell adhesion to the intermediate filament cytoskeleton: direct interaction between the beta4 subunit and plectin at multiple molecular sites. J Cell Biol. 1998;141:209‐225. 9531560 10.1083/jcb.141.1.209 PMC2132717

[44] Garcia‐Alvarez B , Bobkov A , Sonnenberg A , de Pereda JM . Structural and functional analysis of the actin binding domain of plectin suggests alternative mechanisms for binding to F‐actin and integrin beta4. Structure. 2003;11:615‐625. 12791251 10.1016/s0969-2126(03)00090-x

[45] Nievers MG , Kuikman I , Geerts D , Leigh IM , Sonnenberg A . Formation of hemidesmosome‐like structures in the absence of ligand binding by the (alpha)6(beta)4 integrin requires binding of HD1/plectin to the cytoplasmic domain of the (beta)4 integrin subunit. J Cell Sci. 2000;113(Pt 6):963‐973. 10683145 10.1242/jcs.113.6.963

[46] Litjens SH , Wilhelmsen K , de Pereda JM , Perrakis A , Sonnenberg A . Modeling and experimental validation of the binary complex of the plectin actin‐binding domain and the first pair of fibronectin type III (FNIII) domains of the beta4 integrin. J Biol Chem. 2005;280:22270‐22277. 15817481 10.1074/jbc.M411818200

[47] Kostan J , Gregor M , Walko G , Wiche G . Plectin isoform‐dependent regulation of keratin‐integrin alpha6beta4 anchorage via Ca^2+^/calmodulin. J Biol Chem. 2009;284:18525‐18536. 19419971 10.1074/jbc.M109.008474 PMC2709376

[48] Kim TH , Kim HI , Soung YH , Shaw LA , Chung J . Integrin (alpha6beta4) signals through Src to increase expression of S100A4, a metastasis‐promoting factor: implications for cancer cell invasion. Mol Cancer Res. 2009;7:1605‐1612. 19808905 10.1158/1541-7786.MCR-09-0102

[49] Yang X , Dutta U , Shaw LM . SHP2 mediates the localized activation of Fyn downstream of the alpha6beta4 integrin to promote carcinoma invasion. Mol Cell Biol. 2010;30:5306‐5317. 20855525 10.1128/MCB.00326-10 PMC2976378

[50] Liu SY , Ge D , Chen LN , et al. A small molecule induces integrin beta4 nuclear translocation and apoptosis selectively in cancer cells with high expression of integrin beta4. Oncotarget. 2016;7:16282‐16296. 26918348 10.18632/oncotarget.7646 PMC4941314

[51] Frijns E , Kuikman I , Litjens S , et al. Phosphorylation of threonine 1736 in the C‐terminal tail of integrin beta4 contributes to hemidesmosome disassembly. Mol Biol Cell. 2012;23:1475‐1485. 22357621 10.1091/mbc.E11-11-0957 PMC3327322

[52] Lin Y , Rao J , Zha XL , Xu H . Angiopoietin‐like 3 induces podocyte F‐actin rearrangement through integrin alpha(V)beta(3)/FAK/PI3K pathway‐mediated Rac1 activation. Biomed Res Int. 2013;2013:135608. 24294595 10.1155/2013/135608 PMC3835706

[53] Chen Z , Wan X , Hou Q , et al. GADD45B mediates podocyte injury in zebrafish by activating the ROS‐GADD45B‐p38 pathway. Cell Death Dis. 2016;7:e2068. 26794661 10.1038/cddis.2015.300 PMC4816163

[54] Ghosh J , Das J , Manna P , Sil PC . The protective role of arjunolic acid against doxorubicin induced intracellular ROS dependent JNK‐p38 and p53‐mediated cardiac apoptosis. Biomaterials. 2011;32:4857‐4866. 21486680 10.1016/j.biomaterials.2011.03.048

[55] Chen X , Wang L , Zhao Y , et al. ST6Gal‐I modulates docetaxel sensitivity in human hepatocarcinoma cells via the p38 MAPK/caspase pathway. Oncotarget. 2016;7:51955‐51964. 27340870 10.18632/oncotarget.10192 PMC5239527

[56] Asanuma K , Yanagida‐Asanuma E , Faul C , Tomino Y , Kim K , Mundel P . Synaptopodin orchestrates actin organization and cell motility via regulation of RhoA signalling. Nat Cell Biol. 2006;8:485‐491. 16622418 10.1038/ncb1400

[57] Schaldecker T , Kim S , Tarabanis C , et al. Inhibition of the TRPC5 ion channel protects the kidney filter. J Clin Investig. 2013;123:5298‐5309. 24231357 10.1172/JCI71165 PMC3859394

[58] Buvall L , Wallentin H , Sieber J , et al. Synaptopodin is a coincidence detector of tyrosine versus serine/threonine phosphorylation for the modulation of rho protein crosstalk in podocytes. J Am Soc Nephrol. 2017;28:837‐851. 27628902 10.1681/ASN.2016040414 PMC5328162

